# Physiotherapy movement based classification approaches to low back pain: comparison of subgroups through review and developer/expert survey

**DOI:** 10.1186/1471-2474-13-24

**Published:** 2012-02-20

**Authors:** Nicholas V Karayannis, Gwendolen A Jull, Paul W Hodges

**Affiliations:** 1 School of Health and Rehabilitation Sciences, NHMRC Centre of Clinical Research Excellence in Spinal Pain, Injury and Health, The University of Queensland, Brisbane, Qld 4072, Australia

## Abstract

**Background:**

Several classification schemes, each with its own philosophy and categorizing method, subgroup low back pain (LBP) patients with the intent to guide treatment. Physiotherapy derived schemes usually have a movement impairment focus, but the extent to which other biological, psychological, and social factors of pain are encompassed requires exploration. Furthermore, within the prevailing 'biological' domain, the overlap of subgrouping strategies within the orthopaedic examination remains unexplored. The aim of this study was "to review and clarify through developer/expert survey, the theoretical basis and content of physical movement classification schemes, determine their relative reliability and similarities/differences, and to consider the extent of incorporation of the bio-psycho-social framework within the schemes".

**Methods:**

A database search for relevant articles related to LBP and subgrouping or classification was conducted. Five dominant movement-based schemes were identified: Mechanical Diagnosis and Treatment (MDT), Treatment Based Classification (TBC), Pathoanatomic Based Classification (PBC), Movement System Impairment Classification (MSI), and O'Sullivan Classification System (OCS) schemes. Data were extracted and a survey sent to the classification scheme developers/experts to clarify operational criteria, reliability, decision-making, and converging/diverging elements between schemes. Survey results were integrated into the review and approval obtained for accuracy.

**Results:**

Considerable diversity exists between schemes in how movement informs subgrouping and in the consideration of broader neurosensory, cognitive, emotional, and behavioural dimensions of LBP. Despite differences in assessment philosophy, a common element lies in their objective to identify a movement pattern related to a pain reduction strategy. Two dominant movement paradigms emerge: (i) loading strategies (MDT, TBC, PBC) aimed at eliciting a phenomenon of centralisation of symptoms; and (ii) modified movement strategies (MSI, OCS) targeted towards documenting the movement impairments associated with the pain state.

**Conclusions:**

Schemes vary on: the extent to which loading strategies are pursued; the assessment of movement dysfunction; and advocated treatment approaches. A biomechanical assessment predominates in the majority of schemes (MDT, PBC, MSI), certain psychosocial aspects (fear-avoidance) are considered in the TBC scheme, certain neurophysiologic (central versus peripherally mediated pain states) and psychosocial (cognitive and behavioural) aspects are considered in the OCS scheme.

## Background

There is wide variability in the presentation of low back pain (LBP), yet if common characteristics emerge in the assessment that help to distinguish one pain profile from another, they may aid in initial decision-making by defining a dysfunction pattern towards which a targeted intervention is directed. A major goal over several years has been to divide people with LBP into homogeneous populations or 'subgroups' of similar characteristics in an effort to improve patient outcomes [1,2]. Subgrouping may also help reduce inefficient variability in treatment and provide a helpful communication tool [3]. Multiple disciplines have attempted to distinguish LBP subgroups with various classification schemes [4-6]. Perspectives vary, with focus on improving multidisciplinary dialogue [7-9], examination of the musculoskeletal [10-14] or nervous system [15-17], assessment of psychosocial factors [18-25], or attempts to integrate assessment of multiple systems [14,26] to variable degrees. Within physiotherapy, a profession with strong background in neuromusculoskeletal evaluation, classification schemes that focus on directing specific treatment have emerged [10-14] and most include evaluation of the relationship between movement and pain.

Surveys of practice of physiotherapists have revealed low use of classification schemes despite evidence that treatment of patients based on subgrouping results in better outcomes than treatment based on clinical guidelines [27]. Usage rates of 7-70% [28-34] have been reported. The prevalence of relatively low use of these classification schemes could be explained by unfamiliarity with these approaches; low perceived value in classification oriented assessment; inability to choose between classification schemes; or a preference for other assessment methods. An alternative reason for the modest implementation of the classification approach may be that assessment schemes do not adequately integrate the multiple dimensions that can contribute to, or perpetuate LBP. It has been argued that a limitation of schemes used in physiotherapy is the limited consideration of psychosocial aspects of LBP [35]. Given current evidence which suggests subgrouping results in better outcomes than non-subgrouping, and the diversity of schemes with varying 'biological' and 'psycho-social' perspectives, it is timely to compare and contrast the dominant schemes within physiotherapy in terms of their underlying philosophies, what they measure, how they overlap and what, if any, additional factors require consideration.

The aims of this review with developer/expert survey were: (1) To identify the classification schemes designed for use by physiotherapists that include a movement component; (2) To review the theoretical basis and key elements for the schemes with clarification by developers/experts; (3) To review reliability; (4) To identify areas of convergence or divergence between schemes; and (5) To determine how each scheme considers various aspects of the bio-psycho-social framework.

## Methods

**Figure 1:**
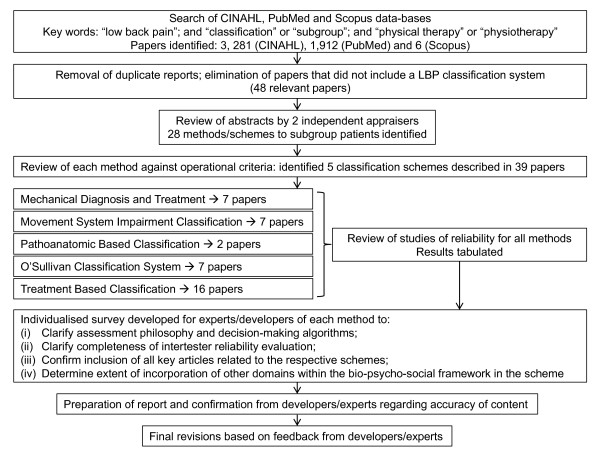
**Study design**.

Literature was reviewed to address the study aims and a two-round survey of the schemes' developers/experts was undertaken to verify accuracy of interpretation of the literature, and to gain their consideration of how their approach addresses broader aspects of a bio-psycho-social framework. Figure 1 depicts the study phases. The initial phase involved systematic search of CINAHL, PubMed and Scopus databases to identify studies pertaining to movement-based physiotherapy classification schemes for low back pain published between January 01, 1985 to June 01, 2011 using the key words: "low back pain"; and "classification" or "subgroup"; and "physical therapy" or "physiotherapy". Articles were required to be written in English. The authors of the manuscript (NK, GJ, PH) developed the search strategy and it was implemented by NK.

The next phase of the review process involved identification of classification schemes that were aligned to operational criteria we developed for movement-based physiotherapy classification schemes for low back pain. We operationally defined a classification scheme as a clinical assessment method that: (i) was applicable to a 'non-specific' LBP group, (ii) identified multiple subgroups within the 'non-specific' LBP population, (iii) included consideration of examination of movement using specific trunk movement tests to discriminate groups, (iv) defined a decision-making algorithm and proposed treatment, (v) was viable within a typical outpatient orthopaedic setting (i.e. did not require electromyography, three-dimensional movement analysis equipment, or statistical software) and (vi) included data on validity and intertester reliability of proposed subgroups. Two independent assessors reviewed the methods used to subgroup patients with LBP that had been identified in the initial phase of the review to determine the alignment of each to these operational criteria. Additional sources of information were used when data were not available from the identified literature. These sources included other publications by experts/developers for each approach (identified by author's name), textbooks, book chapters [10,13,36] and introductory coursework manuals [37-39]. Notes were made regarding the alignment of the schemes against the operational criteria and schemes were included or excluded from further consideration in this review based on the outcome of this phase. Any disagreement between appraisers was resolved through discussion. As viability of subgrouping methods is diminished if it is not consistent between clinicians, additional data was sought for each of the final group of classification schemes regarding reliability and the results tabulated. This phase also involved analysis of the quality of reliability studies according to standardised criteria [40]. Additional file 1 lists the items used in the critical appraisal. Kappa values > 80% were considered to represent excellent agreement; > 60%, substantial agreement; 40-59% moderate agreement; and < 40% poor to fair agreement [41].

An individualised survey was sent to developers/experts (n = 7) for the schemes that satisfied the operational criteria in order to request clarification and further elaboration of any element of the scheme that was unclear from the available literature (i.e. assessment philosophy and decision-making algorithms,), to clarify whether any key articles from their respective schemes were omitted in the initial search strategy and to confirm the completeness and accuracy of the assessment of reliability of the scheme. Developers/experts were also asked to comment on similarities and differences with the other schemes, and to comment on the inclusion of broader bio-psycho-social dimensions within their scheme.

After retrieval of all relevant information from the literature and developer/expert survey responses, the next phase was to review the material for clear areas of convergence and divergence among the 5 classification schemes. Survey results and consideration of convergence and divergence were integrated into the manuscript and the final draft was sent to the developers/experts for final approval of accuracy.

## Results

### Identification of classification schemes

Initial search of the CINAHL, PubMed and Scopus databases identified 3,281 (CINAHL), 1,912 (PubMed) and 6 (Scopus) articles that used the defined keywords, respectively (Figure 1). Removal of duplicate reports and elimination of articles that obviously did not include a LBP classification system left 48 relevant articles. Review of the abstracts by 2 independent appraisers identified 28 methods/schemes to subgroup patients. Review of each of these methods against the operational criteria identified 5 classification schemes, described in 39 articles. The schemes were: Mechanical Diagnosis and Treatment (MDT, McKenzie) [42,43] (7 articles), Treatment Based Classification (TBC, Delitto) [11] (16 articles); Pathoanatomic Classification (PBC, Petersen) [12] (2 articles); Movement System Impairment Classification (MSI, Sahrmann) [44] (7 articles); and the O'Sullivan Classification System (OCS) [45] (7 articles). The 23 schemes/methods that were identified in the initial review, but not included in the final list and the reason for exclusion from the list are presented in Table 1.

**Table 1:** Excluded classification methods

Scheme/developer	Articles	Reason for exclusion from review
Bendebba*	[46]	Relied on spatial distribution of patient's pain and results of straight leg raise test only (does not contain a series of tests and examination of trunk movement)

Bergstrom*	[47]	Used questionnaire to subgroup

Bernard & Kirkaldy-Willis*	[48]	Used a retrospective review of medical records, reliance on radiography, injection, and/or spinal surgery to determine subgroups

Binkley	[49]	Survey which discusses MDT, TBC & MSI schemes

DeRosa & Porterfield*	[50]	Classification based on symptom and history only (i.e., acute injury vs. reinjury vs. chronic pain syndrome), no data on validity or intertester reliability

Halpern*	[51]	Provides a taxonomy of functional assessment constructs linked with the International Classification of Impairments, Disabilities & Handicaps (ICIDH)

Harper*	[52]	Structured according to ICIDH as a conceptual framework

Heinrich*	[53]	Numerical classification system requiring the use of a statistical package

International Classification of Functioning (ICF, World Health Organization)*	[54-58]	Scheme does not discriminate between subgroups based on a defined movement examination system, not suitable for evaluation of responses to treatments [59]

Keefe*	[20]	Observation of motor pain behaviour to distinguish levels of guarding and/or bracing

Kilsgaard	[60]	Article in Dutch language

Klapow*	[23]	Psychosocial factor discrimination only, no analysis of physical impairments

Krause*	[61]	Target population consists of occupational low back pain & describes a phase model of disability

Langworthy & Breen	[62]	Requires a highly standardized computerized interview system, identifies two categories (mechanical and cyclic) with undefined treatment decisions

Laslett & van Wijmen	[63]	Not identified as significantly different than MDT approach, no follow-up validity or intertester reliability studies

MacDonald*	[64]	No validity or reliability studies

Main*	[65]	Used questionnaires to identify level of distress (no movement based examination)

McCarthy et al.	[66]	Review which discusses approaches of Barker, Bendebba et al., Bergstrom et al., Binkley et al., Coste et al., Delitto et al. (TBC), DeRosa & Porterfield, Halpern, Harper et al., Heinrich et al., Humphreys, Huyse et al., Keefe et al., Klapow et al., Krause et al., Langworthy & Breen, Laslett & van Wijmen, MacDonald, Main et al., McKenzie & May (MDT), Moffroid et al., Ozguler et al., Petersen et al., Rezaian et al., Sikorski, Spitzer et al., Strong et al., Van Dillen et al. (MSI), and Wilson et al.

Moffroid*	[4]	Uses questionnaires & physical tests of symmetry, passive & dynamic mobility & strength from the National Institute of Occupational Safety & Health Low Back Atlas (Subgroups: Very Unfit, Unfit, Inflexible, Flexible, Very Flexible) but does not define treatment for proposed subgroups

Newton*	[67]	Treatment decision-making for identified subtypes not defined, provides prevalence but no validity or intertester reliability studies

Ozguler*	[68]	Used response from Dallas Pain Questionnaire only.

Petersen	[69]	Review which discusses MDT, Sikorski, Bernard & Kirkaldy-Willis, QTF, TBC, Newton, Kilsgaard schemes

Quebec Task Force (QTF)*	[70]	Certain categories require advanced imaging, categories not mutually exclusive, undefined treatment for categories

Rezaian*	[71]	Relies on patient history only, defines only two types (constant and intermittent), does not outline treatment for subgroups, no validity or intertester reliability studies

Schäfer et al.	[17,72-75]	Scheme pertains only to low back-related leg pain and hence, not the majority of people with non-specific LBP

Spoto	[76]	Survey which discuss MDT, TBC & MSI schemes

Stiefel*	[59]	Classification relied on response to questionnaire-interview only (INTERMED)

Strong*	[77]	Classification relied on response to a questionnaire-interview only (Integrated Psychosocial Assessment Model)

Wilson	[78]	Philosophical and practical basis derived from the MDT approach with some further category subdivision, not considered significantly distinct from MDT classification system

*Did not meet our operational definition of a classification scheme

### Theoretical basis and key elements of schemes

The conceptual model of each scheme is summarized below. Table 2 lists the subgroups defined in each scheme and the relative prevalence as reported by the developer/expert.

**Table 2:** Classification categories

Classification Scheme	Categories	
Mechanical Diagnosis and Treatment (MDT)	Derangement Syndrome ♦ a. Central & symmetrical b. Unilateral & proximal to knee c. Unilateral & distal to knee Dysfunction Syndrome a. Flexion b. Extension c. Lateral Shift/Side-Gliding d. Adherent Nerve Root	Postural Syndrome ◆ Other a. Stenosis b. Hip c. Sacroiliac joint d. Mechanically inconclusive e. Spondylolisthesis f. Chronic pain state

Treatment Based Classification (TBC)	Specific Exercise a. Flexion b. Extension c. Lateral Shift/Side-Gliding	Manipulation ♦ Stabilization ◆ Traction* (*Prevalence unknown)

Pathoanatomic Based Classification (PBC)	Disc Syndrome ♦ a. Reducible b. Irreducible c. Non-mechanical Nerve Root Compression Spinal Stenosis Zygopophyseal Joint	Postural Sacroiliac Joint Dysfunction Myofascial Pain Adverse Neural Tension Abnormal Pain Inconclusive ◆

Movement System Impairment Syndromes (MSI)	Rotation with Extension ♦ Rotation with Flexion	Rotation Extension Flexion ◆

O'Sullivan Classification System (OSC)	Control Disorder a. Multidirectional ♦ b. Flexion c. Lateral Shift d. Active Extension e. Passive Extension	Movement Disorder a. Flexion b. Extension c. Flexion with Rotation/Side bending d. Extension with Rotation/Side bending Pelvic Girdle Pain ◆ a. Form closure b. Force closure

♦ = Most prevalent*

◆ = Least prevalent* (Based on classification scheme studies and developer survey response)

#### Mechanical Diagnosis and Treatment (MDT)

The primary objective of the MDT scheme is to determine if LBP symptoms can be abolished or reduced through application of direction-specific, repeated lumbar spine movements or sustained postures. Internal intervertebral disc displacement is a key component in the conceptual explanation of this model, particularly for the primary category, "derangement". Less common categories are termed "dysfunction" and "postural". With "dysfunction syndromes" it is thought that tissue has undergone "contraction, scarring, adherence, adaptive shortening, or imperfect repair" [37]. Intervention is based on the tissue remodelling theory. "Postural syndrome" is assumed to arise from joint capsule and ligament ischemia due to prolonged spinal end range positioning. Lifestyle factors believed to predispose individuals to each syndrome include high frequency of sitting, flexion biased posture and activities.

A principal assessment is the use of direction-specific "loading strategies" [79] to elicit a phenomenon termed "centralisation", i.e., movement of peripherally located pain to a more central location. Loading strategies involve repeated end range lumbar extension, flexion or side-gliding movements and are thought to relocate symptomatic displaced tissues in a "derangement syndrome", or stretch adhered or shortened tissue in a "dysfunction syndrome".

#### Treatment Based Classification (TBC)

The primary purpose of the TBC approach is to identify features at baseline that predict responsiveness to four different treatment strategies. Three levels of classification are implemented [11]. The first level determines whether the patient can be managed by physical therapy alone, will require multidisciplinary management, or require referral to another health care practitioner. The second level stages the patient based on the severity of symptoms and degree of disability. Stage I is the acute phase where the goal is symptom relief. Stage II is the subacute phase when symptom relief and quick return to normal function are encouraged. Stage III is for those who must return to high physical demands but demonstrate poor physical conditioning. The third level of classification consists of assignment of the patient to one of four treatment syndromes [11]: "manipulation", "stabilization", "specific exercise" or "traction". Interpretation of a collection of signs, symptoms and observations derives this decision. Signs from four primary orthopaedic tests include: (1) pain centralisation with two or more movements in the same direction, or pain peripheralisation in the opposite direction of centralisation, (2) straight leg raise range of motion, (3) "prone instability test" and (4) lumbar posterior-to-anterior mobility testing. Signs of nerve root compression are identified by a neurologic examination. Symptoms of relevance are: (1) pain duration, (2) pain location, (3) episode frequency and (4) fear avoidance behaviour [11]. Observation is used to detect presence or absence of aberrant motion or a lateral shift deformity. Age is considered a predictor for allocation to the "stabilisation" subgroup.

The "manipulation" subgroup treatment includes high velocity thrust manipulation directed towards the lumbo-pelvic region [11]. The "stabilisation" subgroup receives exercises aimed at "promoting stability by producing motor patterns of co-contraction among all spinal stabilising muscles" [11]. The "specific exercise" subgroup is treated primarily with repeated end range spinal movement in the direction found to elicit a centralisation effect. The "traction" subgroup receives static mechanical traction in prone along with exercises to centralise symptoms [11]. Although initiated as an 'expert panel consensus driven' approach, the TBC scheme has developed into a 'data driven' model, which incorporates evidence from 'clinical prediction rules' [80].

#### Pathoanatomic Based Classification (PBC)

The PBC scheme attempts to connect a symptomatic response to key orthopaedic tests and an assumed pathologic structure to direct treatment [12]. Nine of 11 categories consist of all possible structures that could cause LBP, based on discography and single injection diagnostic studies. The exceptions are "inconclusive" and "abnormal pain syndrome" categories, the latter defined by "abnormal illness behaviour" [81]. Syndromes are defined by symptom location and effect of mechanical loading.

This scheme employs a hierarchy approach in which structures that more commonly cause LBP are considered first [82,83], followed by systematic inclusion or exclusion of less prevalent structures and conditions. Other than response to loading strategies, additional criteria used for diagnosis include: (1) symptom duration; (2) standing, walking, sitting, and lying tolerance; (3) age > 65 years; (4) mechanical aggravating factors; (5) symptom provocation with examination; and (6) "non-organic" signs. "Adherent nerve root" and "nerve root entrapment" categories have been excluded from the system due to low intertester reliability, and the criteria for "spinal stenosis syndrome" and "zygopophyseal joint problem" have been updated (Petersen, survey response).

#### Movement System Impairment Syndromes (MSI)

The major objective in the MSI scheme is to identify the direction of alignment, stress or spinal movement that elicits or increases symptoms [13]. The development of these subgroups is theorized to occur due to alteration in the precision of joint movement as the result of repeated movements and prolonged postures associated with daily activities. This is enhanced by age-related factors such as degenerative changes, habitual postures and repetitive direction-specific activities (Sahrmann, survey response). Prolonged postures and repeated movements are proposed to cause tissue adaptations that eventually result in a joint developing a susceptibility to movement in a specific direction. Stress related to the alteration in precise motion is assumed to cause tissue micro-trauma and eventually, macrotrauma. Repeated movements and associated tissue adaptations are reasoned to contribute to generalization of movement patterns including the imprecise joint motion that is direction-specific. Of particular interest is the concept of relative stiffness that is believed to contribute to and perpetuate a joint's directional movement susceptibility. The relative stiffness problem can pertain to intervertebral joints, as well as other joints such as the hip or shoulder. Studies of the MSI system have documented relative flexibility issues between the lumbar and hip region, where early or excess lumbar motion is observed with lower limb movement tests and with trunk sidebending [84,85].

The MSI scheme focuses on modification of this altered alignment and motion to reduce spine symptoms and to redistribute movement to other joints [86]. This process involves correction and/or restriction of altered lumbar motion in the direction associated with symptoms and facilitation of movement of other joints. Subclassification is confirmed by "modified movement and alignment tests" that correct the impaired spine movement, redistribute motion, or change alignment to reduce or eliminate symptoms. Examination includes tests that "identify tissue adaptations contributing to the identified relative stiffness and associated movement patterns" (Sahrmann, survey response). Tissue adaptations contributing to the patient's altered movement and alignment impairments include motor control alterations in recruitment patterns and timing, de-recruitment, skeletal and muscular performance alterations, stiffness and length. Of critical importance is the biomechanical consideration of muscle performance and recruitment patterns.

#### O'Sullivan Classification System (OCS)

The objective of the OCS is to identify the underlying mechanisms that are considered to drive pain. One of these is the identification of maladaptive (pain provocative) spinal postures, movement patterns, and motor control behaviours associated with LBP, which are then used to target treatment. The development and persistence of these impairments is believed to occur due to a maladaptive response to pain ('movement' or 'control' alteration), resulting in a lack of variance in the way tasks are performed and further perpetuation of pain [14]. Impairments are characterized into either 'pain avoidance' (movement category) or 'pain provocation' (control category) behaviour. Pain response is further categorized as either 'adaptive' or 'maladaptive'. 'Adaptive' pain is considered a protective response secondary to an underlying pathological process, whereas 'maladaptive' pain behaviour is viewed as a process in which movement and cognitive behaviours drive the pain, causing a compromise to the neuromusculoskeletal system. Assessment involves subjective and physical examination of pain, cognitive and movement behaviours [14,36]. The OCS involves five levels [87]: In level one, LBP disorders are separated into 'specific' vs. 'non-specific'. Specific LBP is based on radiological evidence matching the clinical presentation. In level two, the primary pain system is identified as either of a "peripheral" or "central" nervous system disorder. Central nervous system pain disorders (such as regional, neuropathic and fibromyalgia pain disorders) are defined as pain disorders that are constant and non-remitting in nature with no clear mechanical behaviour. Peripheral nervous system disorders refer to localized and anatomically defined pain influenced by mechanical factors, whereas central nervous system disorders refer to constant and widespread pain not clearly influenced by mechanical factors. If the disorder is peripheral, level three involves distinguishing LBP from pelvic girdle pain. Where pain is thought to arise from spinal structures, the fourth level identifies whether "control impairments" (no movement impairment in direction of pain but impaired control) or "movement impairments" (impairment of movement in the direction of pain provocation associated with fear avoidance behaviour) are drivers of the pain disorder. A directional or postural pattern is determined within each group [87]. The fifth level involves identification of psychosocial and/or lifestyle factors that may contribute to the disorder.

Management of non-specific chronic LBP disorders that are considered to be of the peripheral nervous system consist of changing movement and cognitive behaviours based on the disorder classification. "Control impairment" disorders involve enhancing control by training movement patterns in order to functionally unload pain sensitive structures. In contrast, the approach to management of "movement impairment" disorders is to facilitate movement in the direction of pain provocation via graded exposure in order to reduce fear avoidance behaviours. The OCS assesses maladaptive physical and cognitive 'drivers of pain' and implements a behavioural intervention that focuses on the cognitive and behavioural change (termed 'Cognitive Functional Therapy' [36]).

### Review of classification scheme reliability

Quality appraisal scores for each of the intertester reliability articles are reported in Table 3 using the tool devised by Brink and Louw [40]. The MSI scheme demonstrates 'substantial' agreement (Kappa > 60%); the OCS displays 'moderate' to 'excellent' agreement (Kappa 40-80%) dependent on training level; the MDT and TBC schemes show 'moderate' agreement (Kappa 40-60%); and the PBC scheme exhibit 'poor to fair' (Kappa < 40%) intertester reliability. Intertester reliability has been evaluated using various methods, from repeated assessments performed on the same or different day, on-site observation vs. video examination, or written information only. Intertester reliability also differs depending on subgroup, test, and training level (Table 4). The most reliable subgroups across schemes include MDT Derangement, TBC Specific Exercise, PBC Zygopophyseal joint/Dysfunction/Postural syndromes, MSI Flexion & Rotation with Flexion and OCS Control-Passive extension. The most reliable tests across schemes include centralisation (i.e., repeated direction specific movement testing) for both MDT and TBC schemes, and more specifically 'repeated flexion' for the TBC scheme. The most/least reliable test for the PBC, MSI and OCS schemes is unknown. In order to improve MDT "derangement syndrome" reliability, subtypes have been reduced from seven to three. The TBC scheme is the only system with no significant difference between novice (Kappa 0.44-0.76) and expert clinicians (Kappa 0.52-0.87) [88]. The OCS [87] scheme intertester classification reliability improves with training.

**Table 3:** Intertester reliability of the whole classification system

Classification scheme	Study reference	Percentage agreement (Mean)	Kappa value	Confidence interval	**Study quality score **[40]
Mechanical Diagnosis & Treatment (MDT)	Clare [89,90]	91% (main syndrome), 76% sub-syndrome	0.56 (main syndromes), 0.68 (sub syndromes)	0.46-0.66 (main syndrome); 0.67-0.69 (sub-syndrome)	7/13 (5 questions N/A)
	Kilby [91]	58-74%	---	---	9/13 (3 questions N/A)
	Kilpikoski [92]	74-95%	0.6-0.7	---	11/13 (2 questions N/A)
	Razmjou [93]	93-97%	0.7-0.96	---	11/13 (2 questions N/A)
	Riddle [94]	39%	0.26	---	11/13 (2 questions N/A)

Treatment Based Classification(TBC)	Brennan [95]	83%	---	---	7/13 (5 questions N/A)
	Fritz [96]	65%	0.49-0.56	---	9/13 (4 questions N/A)
	Fritz [88]	76%	0.60	0.56-0.64	9/13 (4 questions N/A)
	Heiss [97]	31%-55%	0.14-0.45	---	10/13 (3 questions N/A)
	Henry [98]	79%-81%	0.60	0.56-0.63	Not score- able (No full-text article/conference abstract)

Pathoanatomic Based Classification (PBC)	Petersen [99]	39-72%	0.44-1.0	---	11/13 (2 questions N/A)

Movement System Impairment Syndromes(MSI) **Classification scheme** Movement System Impairment Syndromes (MSI)	Harris-Hayes [100]	83%	0.75	0.51-0.99	11/13 (2 questions N/A)
	Henry [101]	90%	0.81	0.78-0.83	Not score- able (No full- text article/conference abstract)
	Norton [102]	78%	0.57	---	9/13 (2 questions N/A)
	Trudelle-Jackson [103]	75%	0.61	0.33-0.89	11/13 (2 questions N/A)

O'Sullivan Classification System (OSC)	Dankaerts [104]	70-97%	0.61-0.96*	---	11/13 (2 questions N/A)
	Vibe Fersum [105]	73-92%	*Control subgroups only 0.66-0.90	---	10/13 (2 questions N/A)

*N/A denotes critical appraisal question was not applicable to the study design

**Table 4:** Variability in intertester reliability between subgroups and tests

Classification scheme	Subgroup & Test Variables*	Percentage agreement	Kappa value	Confidence interval
Mechanical Diagnosis and Treatment	Most reliable subgroup-Derangement		0.96	---
	Least reliable subgroup-Unknown		---	---
	Most reliable test-Centralization		0.51-0.96	---
	Least reliable test- Lateral shift		0.52	---

Treatment Based Classification*	Most reliable subgroup-Specific exercise	95%	---	---
*Traction subgroup excluded in all cited studies	Least reliable subgroup-Stabilization	64%	---	---
	Most reliable test-Repeated flexion		0.46	---
	Least reliable test-Aberrant motion		0.18	---

Pathoanatomic Based Classification	Most reliable subgroup-Zygopophyseal joint syndrome, Dysfunction syndrome, postural syndrome	100%	1.00	1.00 to 1.00
	Least reliable subgroup-Myofascial pain (MFP) syndrome and other	MFP = 74%	MFP = 0.44	MFP = 0.25 to 0.64
	Most and least reliable test-Unknown	Other = 82%	Other = 0.32	Other = 0.07 to 0.58
		---	---	---

Movement System Impairments	Most reliable subgroup-Flexion, Rotation with flexion	100%	---	---
	Least reliable subgroup-Rotation	84%	---	---
	Most reliable test-Unknown			
	Least reliable test-Unknown			

O'Sullivan Classification Scheme	Most reliable subgroup-Control-passive extension	100%	---	---
	Least reliable subgroup-Control-Active extension	50%	---	---
	Most and least reliable test-Unknown	---	---	---

*Subgroup = the classification category; Test = the observation, movement, or symptomatic response which is used to help distinguish the various subgroups

### Convergence or divergence between schemes

#### Philosophies

All schemes share the objective to identify directions, movements or control patterns that decrease or increase pain in order to direct treatment. In this respect, we considered that the five schemes could be clustered into two groups. One group (MDT, TBC, and PBC) uses the centralisation phenomenon to guide categorization, but schemes vary in loading dosage, assessment strategies, and intervention options. The other group (MSI and OCS) shares a parallel theme of 'modified' movement and alignment strategies and a common philosophy of promoting variety and precision of movement, although the proposed mechanism, movement observation focus, and emphasis on identification of concurrent psychosocial factors differ.

#### Assessment methods

The MSI and OCS schemes rely on identification of the symptomatic region or segment during functional lumbo-pelvic and thoraco-lumbar movement tests and are alike in their use of a variety of test positions. MSI tests focus on the influence of hip and pelvic movements on the lumbar spine in both weight bearing and non-weight bearing positions, whereas OCS tests focus on abnormal thoraco-lumbar and lumbo-pelvic movement primarily in weight bearing positions, determined by pain provocative activities reported by the patient.

Differences exist between the two clusters of schemes in frontal plane analysis, with an assessment preference for lumbar spine side gliding (i.e. lateral pelvic translation) (MDT, PBC, and TBC) versus side bending (MSI and OCS). Lumbar rotation is a key MSI assessment component and part of the OCS multi-directional movement categories, but not referred to in other schemes.

#### Patient appropriateness

The primary reason to implement a classification process for non-specific LBP is to facilitate the 'clustering' of signs and symptoms in order to help better direct treatment and improve efficiency of care. However, the application of the subgrouping systems is also likely to apply to specific LBP diagnoses. The application to patients with specific diagnoses has not been tested, nor is it clear whether having a specific 'pathoanatomic' LBP diagnosis facilitates treatment decisions better than an assessment based on the patient's presentation of movement, pain processing, and emotions, cognitions, and behaviours related to their LBP.

For development and reliability purposes, most studies of classification schemes exclude people with diagnoses such as severe kyphosis, scoliosis, ankylosing spondylitis, and rheumatoid arthritis. Spinal stenosis is integrated as a subgroup within some schemes (MDT and PBC) whereas it is considered a specific diagnosis in the OCS and requires further classification. Spinal stenosis is most often classified in the MSI "extension" or "extension-rotation" subgroup, or TBC "specific exercise--flexion" subgroup (Sahrmann and Fritz, survey responses). The OCS scheme considers disc prolapse with radicular pain, spondylolisthesis, degenerative disc disease with positive Modic changes [106] and positive neural provocation tests as specific LBP diagnoses and are assessed and classified within the scheme. The MSI and OCS scheme developers clarified that principles of the classification approach could be applied to the specific LBP population [39], but require a 'modified approach' (Sahrmann and O'Sullivan, survey responses), taking into account the known pathological influences on the condition. How this is operationalized is less defined.

#### Distinguishing 'mechanical' LBP and 'stage' of disorder

Most schemes are designed for those with 'mechanical' LBP, determined from history, and confirmed with physical examination. It is argued that if there is consistency in symptom provocation that can be related to, or altered by application of certain movements or postures, it is deemed a mechanical problem. Although TBC scheme reliability studies have generally targeted the 'acute' (< 3 months duration) LBP population, and MSI and OCS scheme studies have primarily used a 'chronic' (> 3 months) LBP population, scheme developers/experts consider their scheme appropriate across the LBP stage continuum (all developers/experts, survey response). Table 5 summarises some of the key proposed overlap between subgroups identified within different schemes.

**Table 5:** Proposed subgroup overlap

Flexion oriented provocation	Rotation/Side-bending/Side-gliding oriented provocation	Extension oriented provocation
MDT-Derangement (posterior) or Dysfunction (flexion) or Postural	MDT-Derangement (lateral shift) or Dysfunction (lateral shift)	MDT-Derangement (anterior) or Dysfunction (extension) or Spondylolisthesis
PBC-Disc syndrome or Postural or Dysfunction (flexion)	PBC-Disc syndrome (lateral shift) or Nerve root compression or Dysfunction (lateral shift)	PBC-Disc syndrome or Nerve root compression or Spinal stenosis or Zygopophyseal joint or Dysfunction (extension)
TBC-Specific exercise (extension) or Stabilisation or Manipulation or Traction	TBC-Specific exercise (side-glide) or Manipulation or Stabilisation	TBC-Specific exercise (flexion) or Stabilisation or Manipulation
MSI-Flexion	MSI- Rotation, Rotation with Flexion or Extension	MSI-Extension
OCS-Control-Flexion or Movement-Flexion	OCS-Control-Lateral shift or Multidirectional or Movement-Flexion or Extension with Rotation/Side bending	OCS-Control-Active or Passive Extension or Movement-Extension

### Consideration of the bio-psycho-social framework within the classification schemes

In addition to the biomechanical system influences on LBP, psychosocial and neurophysiologic systems are equally important to consider in a multidimensional LBP assessment model [107-109]. Both psychosocial factors (historical events/past experience with pain/perception of the present injury seriousness, work/family/practitioner environment, culture, personality, appraisals, self-efficacy, emotions/mood, educational and socioeconomic levels, sleep disturbance) and neurophysiologic factors [110] (e.g., pain physiology) potentially influence the movement presentation and frame the pain experience [111-115] and are likely to be important to consider as moderators of the pain presentation and influence treatment selection. This section focuses on how current classification schemes integrate these aspects in assessment and attempt to distinguish psychosocial domains and neurophysiologic pain mechanisms within the assessment strategy.

#### Incorporation of psychosocial factors

As psychosocial factors may contribute to or perpetuate LBP, it can be argued that they cannot be separated from a biomechanical view of LBP. All schemes consider psychological influences, particularly when psychological signs appear dominant in persistent LBP. However, there is disparity across schemes in identification of psychosocial aspects within movement-based categories.

MDT and PBC schemes identify subgroups with an 'abnormal or chronic pain state' by testing for Waddell's 'abnormal illness behaviour' signs [81] but prioritize treatment of the mechanical presentation first. "Chronic pain" is defined by the MDT and PBC schemes as symptoms that are persistent (i.e., > 12 weeks), widespread and increase with all activity. Patients demonstrate "exaggerated pain behaviour and mistaken beliefs and attitudes about pain and movement" [10]. The MDT rationale is that many psychological factors improve when the mechanical presentation and associated pain improves (Clare, survey response). PBC identifies depression as a key predictor of poor recovery and other psychosocial dimensions as poor prognostic indicators such as distress, job satisfaction, duration of sickness, unemployment and financial incentives (Petersen, survey response). How these variables are identified and integrated with the mechanical diagnosis categories is not specified.

Fear-avoidance is the key psychological feature addressed in the TBC scheme, as a low Fear Avoidance Beliefs Questionnaire (FABQ) score is a criterion for allocation to the "manipulation" subgroup. The classification approach can still be applied to those with a high FABQ, but with modification (Delitto, survey response). Likewise, the MSI scheme incorporates a measure for fear avoidance behaviour, but the developers/experts state this population subtype is atypical within their research and clinical setting (Sahrmann and Van Dillen, survey response). How the MSI scheme would address patients who present with high fear avoidance behaviour, or other psychological features, is not clear. The OCS advocates multidisciplinary management and functional rehabilitation in the presence of dominant psychosocial factors [87]. In the presence of non-dominant psychosocial traits, the OCS also integrates the assessment of psychosocial factors into decision-making and pursues a behavioural intervention.

#### Incorporation of neurophysiologic factors

The International Association for the Study of Pain has defined neuropathic pain as "pain initiated or caused by a primary lesion or dysfunction in the nervous system". In an effort to distinguish neuropathic pain from nociceptive system hyperexcitabiity (i.e., central sensitization) [116] a more precise definition of neuropathic pain has been proposed as "pain which arises as a direct consequence of a lesion or disease affecting the somatosensory system" [117]. Where the nociceptive system generates activity without adequate peripheral sensory ending activation, correlates of the neuroanatomic distribution, pain consistency, stimulus-response proportion, history of injury, and findings from a clinical neurologic examination may help to distinguish the likelihood of neuropathic versus nociceptive pain origin. Anatomic location of the lesion/disease can also help to divide neuropathic pain into peripheral and central nervous system subtypes. Recent studies suggest prevalence of neuropathic pain in patients with low back and leg pain is 37% [15] to 41% [118] and is characterised by certain signs, such as radicular pain, higher ratings of pain intensity, depression, panic, anxiety and sleep disorders [119]. LBP patients presenting with neuropathic features tend to have poorer outcomes, hence, consideration of LBP states beyond subcategories of nociceptive (mechanical) or inflammatory (chemical) pain in favour of considering LBP as a 'nervous system/neurodegenerative disease' [120] may provide more comprehensive pain assessment and treatment. It should be acknowledged that neuropathic pain could co-exist with other pain types (i.e., mechanical or chemical), and central sensitization (amplification of transmission pathways within the central nervous system) is one mechanism that can contribute to neuropathic pain. Deciding which pain subtype is more important or dominant is based largely on clinical judgement.

Although most classification schemes provide a biomechanical or presumed pathoanatomic explanation for the presence of pain, few attempt to distinguish between various pain mechanisms. The OCS differentiates pain systems into either a centrally mediated or peripherally mediated pain category [39]. These features are distinguished from one another within the classification process by clinical expert judgment based on a patient's pain behaviour. The "abnormal pain state" subgroup proposed by the MDT and PBC schemes potentially capture some neuropathic characteristics and the PBC scheme provides an "adverse neural tension" category which may further highlight a particular neuropathic subset. However, these categories imply the pain states are separate and do not co-exist or interrelate with more mechanically based subgroups.

## Discussion

Five movement based classification schemes were identified and, from a broad perspective, the theoretical basis and key elements can be generalised into two main approaches. One approach (MDT, TBC, and PBC) is initially guided by evaluation of the response to loading the spine in different directions, and the other (MSI and OCS) is guided by identification of strategies of modified movement along with a process of diagnostics. Inter-tester reliability across schemes varies depending upon the subgroup and level of training, with ranges from 'poor to fair' (PBC), 'moderate' (MDT, TBC, OCS), 'substantial' (MSI), and 'excellent' (OCS).

Several areas of convergence and divergence were identified between schemes. Most share a common clinical reasoning strategy to classify patients into subgroups based on relevance of a specific movement direction to the symptoms in order to direct intervention and predict outcomes. The MDT, TBC and PBC schemes also offer categories for patients who do not fit into a directional preference. MDT, TBC, and PBC schemes converge in their use of repeated spinal movements to investigate the phenomenon of centralisation of pain, but diverge in their relative emphasis on this parameter, the inclusion of additional assessment options, and differences in the recommended treatments. The MSI and OCS schemes share the use of modification of painful movement to aid allocation to a subgroup, but they diverge in their emphasis on impairments, the specific clinical tests used, and the relative emphasis on neurophysiologic and psychosocial factors. Differences between classification philosophies and strategies are greater than similarities. Disparity clearly exists between pursuit of a pathoanatomical source of pain (PBC) versus the disengagement from this model (all other schemes). The MDT scheme places emphasis on ignoring known structural pathology and assesses initial pain response to movement in an effort to determine if the centralisation phenomenon can be elicited. The MSI scheme would consider this an inappropriate strategy for a spinal stenosis condition and the TBC scheme would more likely cease provocative movement testing to determine if manipulation or stabilisation exercises would be of benefit. MDT, TBC, MSI, and OCS schemes are designed around a preferred treatment strategy for each subgroup; however, the PBC scheme deliberately avoids selection of treatment.

Diversity exists across schemes in the extent of their consideration of the biopsychosocial framework. In regards to the psychosocial aspects of LBP, the current emphasis in the majority of schemes (MDT, PBC, and TBC) has been to focus on "magnified illness behaviour" [121] and the "fear-avoidance" [111] model to assess the influence of psychological factors in LBP. The OCS scheme appears to integrate a wider psychological spectrum of the attention, cognitive, beliefs and behavioural aspect of LBP. This viewpoint is based on previous OCS studies, which have used the Tampa Scale for Kinesiophobia [122], Örebro Musculoskeletal Pain Screening Questionnaire, Hopkins Symptoms Check List, and FABQ [87], in addition to the scheme's emphasis on incorporating a biopsychosocial model. There is currently a divergence in opinion on how to address psychosocial aspects of LBP. MDT and PBC schemes preferentially treat the mechanical dysfunction regardless of psychological presentation, with the intention that improvement in symptoms may positively affect the psychological domain [123]. The TBC scheme focuses on one behavioural dimension of pain to guide assessment. The OCS scheme attempts to address the cognitive and behavioural aspects of LBP. Future research could explore which approach best reduces persistent or recurrent pain, or if additional psycho-social dimensions should be assessed (i.e., happiness [124], optimism [125], self-efficacy [126], stress hardiness [127], sense of coherence [128], treatment expectancy [129], life satisfaction [130], mindfulness [131]) from both patient and practitioner [132] perspectives.

Further diversity exists across schemes in the extent of their consideration of the neurophysiological aspects of pain within the biopsychosocial framework. Although the MDT scheme acknowledges a "chronic pain state" category, the definition of this subgroup pertains primarily to dominance of psychological factors and less on pain systems theory. For example, if a mechanical approach did not decrease fear avoidance, then a graded exercise intervention would be applied. The PBC scheme may hold a broader perspective of altered sensory features, by including "abnormal pain state" and "adverse neural tension" categories. The OCS scheme separates pain systems into "centrally mediated" and "peripherally mediated" subgroups, although operational criteria require development. How these subgroups relate to a proposed neuropathic pain grading system [117] remains unknown.

Clinical trials are required to validate the use of subgrouping in low back pain. Although additional work is required to determine the optimal sequence of trials to be conducted, at minimum randomised controlled trials are required to determine whether classification of patients on the basis of these schemes leads to better outcomes for people with low back pain. Additional trials are necessary which investigate the relative importance of different aspects of the schemes for treatment outcome (e.g. consideration of movement vs. psychological perspectives).

## Conclusions

Perhaps one reason for the variety of subgrouping schemes lies in the rationale that one assessment method cannot be applicable to all types of patient characteristics, or adequately capture the diverse pool of responses from a single assessment strategy. In contrast, the broad selection of schemes may symbolize a beneficial diversity in assessment viewpoints, or hold insight on proficient and deficient elements within classification systems. It would be of interest to examine which patient profiles respond more favourably to a 'repeated movement' approach or a 'modified movement approach'.

The question has been raised as to whether existing schemes adequately identify the presence of neuropathic pain, which may have implications in terms of seeking the appropriate intervention. Contemporary pain models suggest that different neurophysiologic pathways can distort the perception of pain. Although patients experiencing LBP can be focused on 'what structure' is causing their pain and clinicians can feel compelled to provide patients with a structurally based answer, this often poses a diagnostic dilemma. Broadening the question to 'what processes' are causing the LBP, for example, through neuroscience pain based [133], contextual cognitive behavioural therapy [111,134], mindfulness based stress-reduction [131], or biomechanically but less pathoanatomically focused oriented education may provide additional benefit. Future studies could address whether early identification and targeted education and treatment of neurophysiologically based pain subtypes, or matching a patient's psychological characteristics with the various education styles improves outcomes.

## Competing interests

The authors declare that they have no competing interests.

## Authors' contributions

NK, GJ, and PH have been involved in drafting, revision, and final approval of this manuscript.

## Pre-publication history

The pre-publication history for this paper can be accessed here:

http://www.biomedcentral.com/1471-2474/13/24/prepub

## Supplementary Material

Additional file 1 **Appendix A**. Critical appraisal tool for validity and reliability studies of objective clinical tools, *Items related to reliability (adapted from Brink & Louw, 2011). Click here for file
