# Supplementary material for: Physiotherapy movement based classification approaches to low back pain: comparison of subgroups through review and developer/expert survey
Source: BMC Musculoskelet Disord. 2012 Feb 20;13:24. doi: 10.1186/1471-2474-13-24 (PMC3395852; doi:10.1186/1471-2474-13-24)
Supplement: Additional file 1 — Appendix A. Critical appraisal tool for validity and reliability studies of objective clinical tools, *Items related to reliability (adapted from Brink & Louw, 2011). [file 1471-2474-13-24-S1.DOCX]

**Appendix**

A. Critical appraisal tool for validity and reliability studies of objective clinical tools, *Items related to reliability (adapted from Brink & Louw, 2011)

| **Item** |
| --- |
| 1. If human subjects were used, did the authors give a detailed description of the sample of subjects used to perform the (index) test?* |
| 2. Did the authors clarify the qualification, or competence of the rater(s) who performed the (index) test?* |
| 3. Was the reference standard explained? |
| 4. If interrater reliability was tested, were raters blinded to the findings of other raters?* |
| 5. If intrarater reliability was tested, were raters blinded to their own findings of the test under evaluation?* |
| 6. Was the order examination varied?* |
| 7. If human subjects were used, was the time period between the reference standard and the index test short enough to be reasonably sure that the target condition did not change between the two tests? |
| 8. Was the stability (or theoretical stability) of the variable being measured taken into account when determining the suitability of the time interval between repeated measures?* |
| 9. Was the reference standard independent of the index test? |
| 10. Was the execution of the reference standard described in sufficient detail to permit its replication?* |
| 11. Was the execution of the reference standard described in sufficient detail to permit its replication? |
| 12. Were withdrawals from the study explained?* |
| 13. Were the statistical methods appropriate for the purpose of the study?* |
